# Levodopa induces thyroid function regulation in a patient with thyroid hormone resistance and Parkinson’s disease: a case report

**DOI:** 10.3389/fendo.2025.1536372

**Published:** 2025-03-18

**Authors:** Gabriela Rozo-Paz, Clara Maria Ruiz-Forero, José David Suárez-Mera, Guillermo Monsalve Duarte, William Kattah Calderón

**Affiliations:** ^1^ Universidad de Los Andes, Bogotá, Colombia; ^2^ Universidad Cooperativa de Colombia, Villavicencio, Colombia; ^3^ Fundación Santa Fé de Bogotá, Bogotá, Department of Pathology, Bogotá, Colombia; ^4^ Fundación Santa Fé de Bogotá, Bogotá, Department of Neurological Surgery, Bogotá, Colombia; ^5^ Fundación Santa Fé de Bogotá, Department of Endocrinology, Bogotá, Colombia

**Keywords:** thyroid hormone resistance, dopamine, Parkinson’s disease, levodopa, case report

## Abstract

**Introduction:**

Thyroid hormone resistance (THR) is a rare genetic syndrome characterized by reduced sensitivity to thyroid hormones. Patients may be asymptomatic, although clinical manifestations depend on the THR subtype. This entity commonly has abnormal thyroid function tests and can be confirmed by molecular analyses.

**Case presentation:**

The present study describes a 55 year-old female diagnosed with surgically resected papillary thyroid carcinoma. During the endocrinology consults, elevated thyroid hormone levels were detected without an adequate TSH response, and THR was suspected. Moreover, Parkinson’s disease was diagnosed, and treatment with levodopa/carbidopa was initiated. Following this regimen, her TSH and total T3 levels were subsequently normalized, which suggests a potential effect of this agent on the normalization of these hormone levels in the blood. In this case, the role of levodopa was crucial to regulate the TSH concentration which was required to carry out the resection of a tumoral remnant.

**Conclusion:**

The influence of dopamine in the endocrine system, specifically in the thyroid gland, is beneficial in conditions such as THR where abnormal TSH levels can be lowered, helping to balance the thyroid and hormones function.

## 1 Introduction

Thyroid hormone resistance (THR) or thyroid hormone action defects (THADs) are defined as genetic syndromes characterized by reduced sensitivity to thyroid hormones (1) and are typically associated with mutations in the THRB gene. Its prevalence varies between one in 19,000 and 40,000 live births (2). Clinical manifestations vary depending on the type of THR, and can involve neurological, cardiac, gastrointestinal, and bone discrepancies. Diagnosis involves molecular and thyroid function tests. In this study, we report the case of a 55-year-old woman diagnosed with papillary thyroid carcinoma, THR, and Parkinson’s disease, who was treated with levodopa/carbidopa. Following this regimen, her TSH levels normalized, suggesting the potential effect of this agent on thyroid function.

## 2 Article types

### 2.1 Case presentation

A 55-year-old female patient with a medical history of rheumatoid arthritis and uterine myomatosis was diagnosed with a thyroid gland calcification. Thyroid biopsy was performed, and papillary thyroid carcinoma was detected. Total thyroidectomy was performed with positive resection margins and no signs of lymph node invasion. Radioactive iodine ablation was performed, and further imaging did not show signs of metastases. Thyroid hormone replacement was initiated, and regular follow-up was performed (with no abnormalities), which included thyroid ultrasound, thyroglobulin, and antithyroglobulin antibodies.

**Figure 1:**
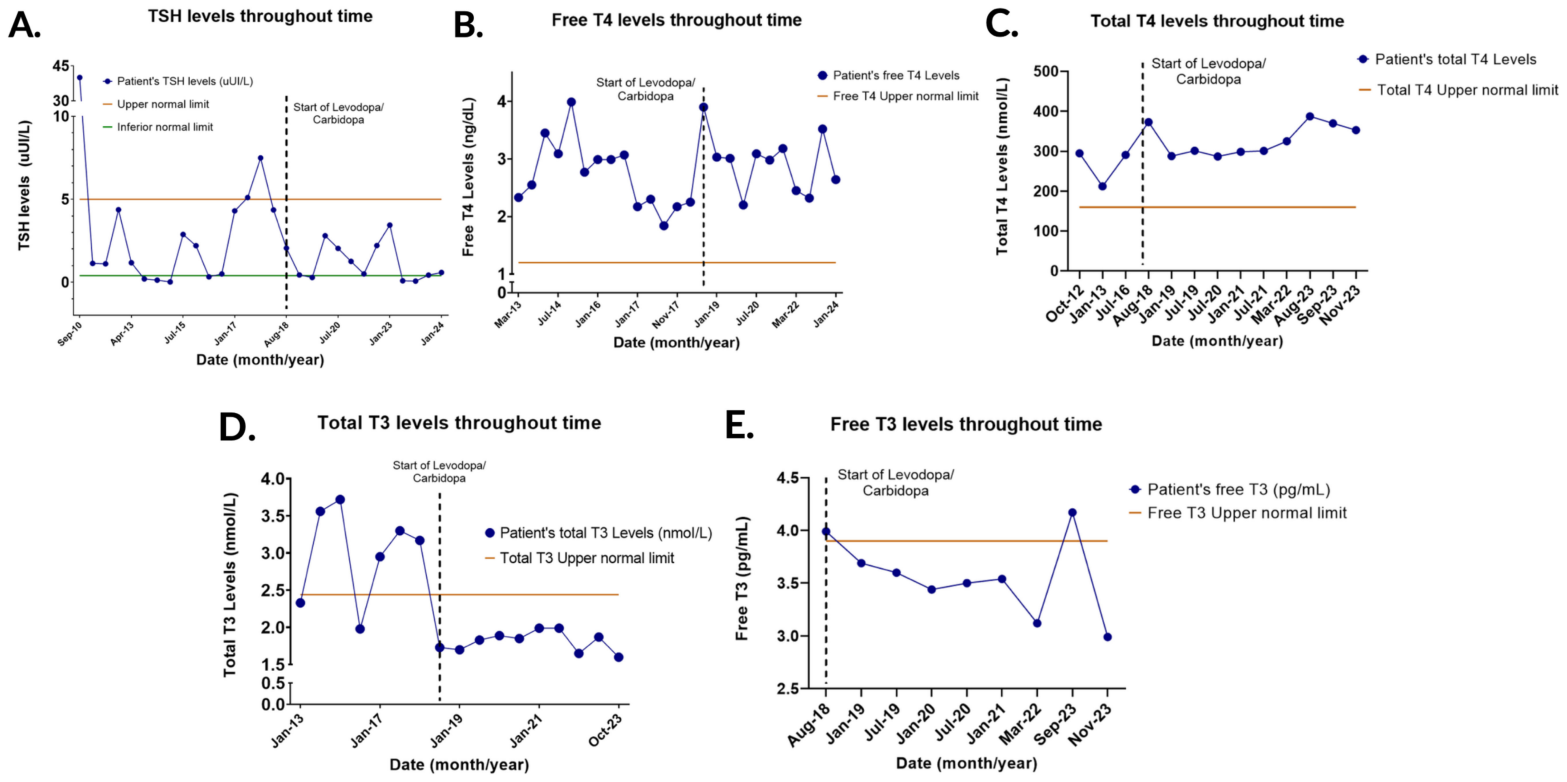
Thyroid hormone levels (TSH, Free T4, Total T4, Total T3, and Free T3) over time. **(A)** TSH levels throughout time. TSH remained mostly in the normal range but managed to stay within the normal range and suppressed with the start of the medication. **(B)** Free T4 levels throughout time. The concentrations remained elevated despite the start of Levodopa/Carbidopa. **(C)** Total T4 levels throughout time. The concentrations remained elevated despite the start of the medication. **(D)** Total T3 levels throughout time. The concentrations normalized when Levodopa/Carbidopa was started. **(E)** Free T3 levels throughout time. Free T3 remained mostly in the normal range and there was no clear change in the TSH levels with the start of Levodopa/Carbidopa.

In the following years, TSH and thyroid hormone levels began with unusual pattern concentrations that continued for almost 7 years. TSH varied in most measurements between the normal range (0,02-40 uUI/L), but total T4 (212-295 nmol/L), free T4 (1,84-3,99 ng/dL), and total T3 (1,98-3,72 nmol/L) were consistently high or in the upper normal limit (**Figures 1A-D**).

**Figure 2:**
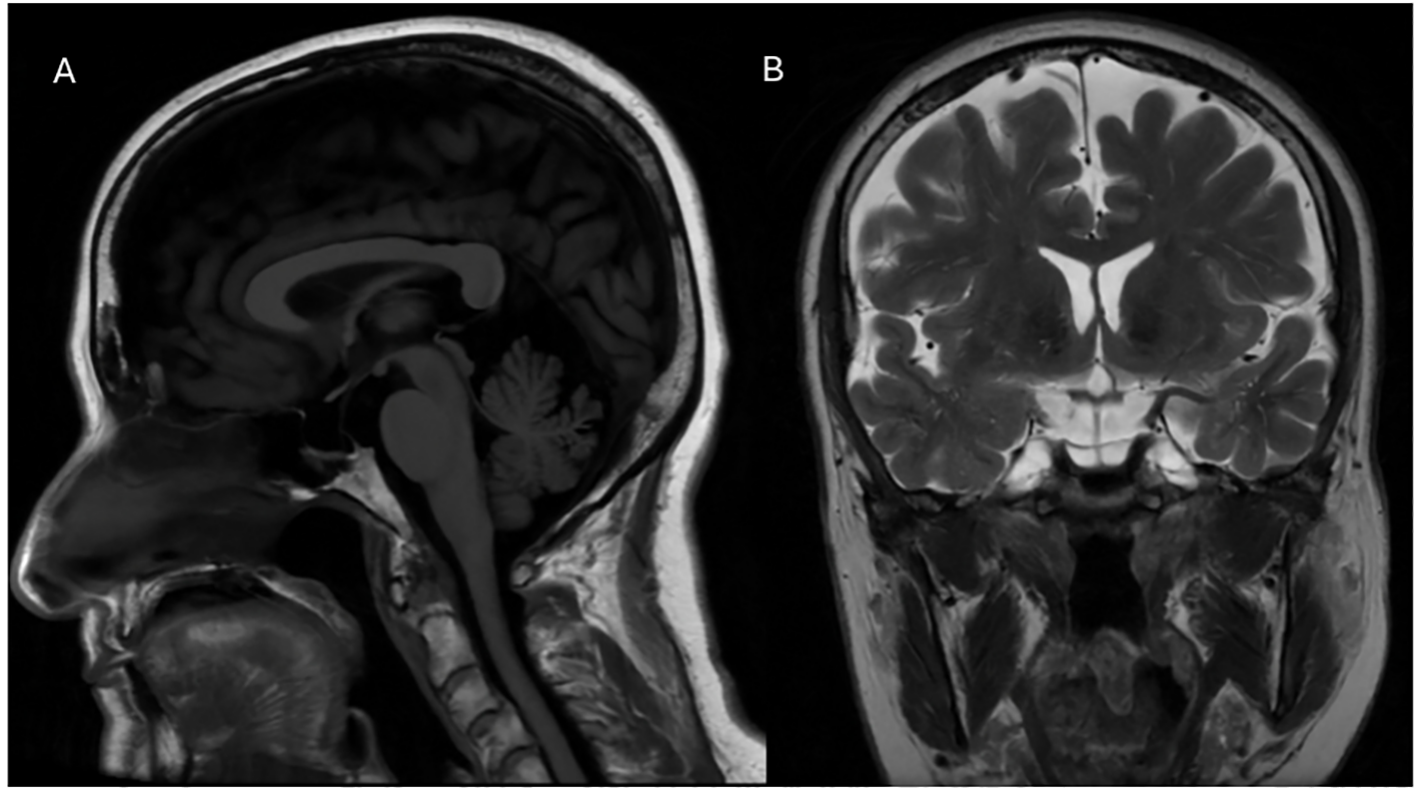
**(A)** Sagittal T1-weighted brain magnetic resonance imaging (MRI) sequence showing the findings of an partially empty sella turcica. **(B)** Coronal T2-weighted MRI sequence confirming the previously described findings.

Subsequently, symptoms such as right hemibody tremor, rigidity, and hypokinesia were observed, and Parkinson’s disease was diagnosed. Initial treatment consisted of selegiline and pramipexole. Additionally, TSH levels increased above the upper normal limit. Therefore, a sella turcica MRI was performed finding a partial empty sella (**Figures 2A, B**) and alpha-subunit was found within normal limits, ruling out a pituitary adenoma. Parkinson’s symptoms continued to progress with a wide stepping gait, cogwheel rigidity, and tremor at rest in the right hand. Consequently, selegiline was replaced by rasagiline, and rotigotine was added; however, the latter was discontinued due to the emergence of a papular rash in the area of administration. Levodopa/carbidopa was started because of the worsening of symptoms and thyroid function tests showed TSH (0,29-3,44 uUI/L), total T3 levels (1,65-1,99 nmol/L) and free T3 (3,12-3,99 pg/mL) within normal limits. Nevertheless, total T4 (287-373 nmol/L) and free T4 levels (2,2-3,9 ng/dL) steadily increased (**Figures 1A-E**).

Recently, the patient presented with hyperphagia and overspending, which was probably related to pramipexole. The medication was discontinued, and safinamide was added. In the latest medical follow-up, a thyroid ultrasound was performed, and lymph node mapping detected a nodular lesion in the thyroid bed measuring 8.8 x 7.3 millimeters. Moreover, thyroglobulin levels rose from 1 to 4 uUI/L in the last year. Biopsy confirmed the presence of atypical follicular cells. Thyroid function tests were abnormal with suppressed TSH (0,09 uUI/L), normal total T3 (1,87), and elevated free T3 (4,17 pg/mL) and total T4 (387,6 nmol/L) (**Figures 1A, C-E**). Thyroid hormone replacement was adjusted, and TSH concentration was later normalized.

The patient underwent lymph node resection, which was positive for malignancy. In the upcoming weeks, radioactive iodine will be administered. Over the years, thyroid function tests showed that free T4 and total T4 levels steadily increased, while TSH was mostly high or within the normal limits, suggesting a possible thyroid hormone resistance syndrome. Moreover, total T3 levels were abnormally elevated until levodopa/carbidopa was initiated, and the concentrations decreased below the upper normal limit. TSH levels also decreased without rising again above the upper normal limit, indicating a possible influence of levodopa on thyroid function.

### 2.2 Discussion

#### 2.2.1 Hypothalamic-pituitary-thyroid axis and thyroid hormone resistance

Thyroid function operates through various integrated mechanisms starting at the hypothalamic paraventricular nucleus which synthesizes thyrotropin-releasing hormone (TRH). It is then transported to the anterior pituitary gland where it promotes the transcription of thyroid-stimulating hormone (TSH) and prolactin. Moreover, TSH stimulates the production of thyroxine, tetraiodothyronine (T4), and triiodothyronine (T3) in the thyroid gland. Deiodinases transform T4 into T3 or inactive reverse T3 in peripheral tissues (3).

In most cases, T3 interacts with thyroid hormone receptors, which are transcription factors that encourage oxygenation, expression of beta receptors in cardiac tissue, and increase metabolic rate and skeletal growth (3, 4). These receptors are classified as TRα and TRβ, which are encoded by THRA and THRB respectively (5). Nevertheless, this process can be altered, causing THR. This group of inherited disorders can be classified into three subtypes: alterations in the conversion of T4 to T3 by deiodinases, abnormalities in the cellular transport of thyroid hormones, and mutations in thyroid hormone receptors (1)​. The latter is also known as classical THR, and the non-classical form can be caused by mutations in proteins such as DIO1 and SBP2, which alter the conversion of thyroxine to triiodothyronine and proteins such as MCT8 and OATP1C1, which normally contribute to the transport of thyroid hormones (5)​. THR with a mutation in the beta receptor is clinically characterized by hyperactive behavior, goiter, attention deficit, tachycardia, and hearing alterations (4, 5)​​. The variation in the SBP2 protein causes a delay in bone development and low stature and the absence of MCT8 can cause axial hypotonia, spasticity of the lower extremities, and diminished verbal communication. The definitive diagnosis is made through genetic testing, but it can be suspected by thyroid function tests that tend to have elevated T3, T4, and TSH levels in the classical form of THR (1)​. Nevertheless, it has been described that in 15% of cases of RHT, the mutated receptor gene is not identified, and it is thought to be associated with proteins or other factors that affect the thyroid hormone receptor in a direct or indirect way (1)​. The purpose of THR treatment is to maintain an adequate TSH level. Antithyroid agents, thyroid hormones, and TH analogs such as triiodothyroacetic acid or β-adrenergic blockers can be administered, depending on the patient’s clinical features (4, 6).

#### 2.2.2 Empty Sella turcica findings and its correlation with thyroid hormone resistance

Empty sella is usually a neuroradiological finding characterized by an apparently empty sella turcica, where the pituitary gland appears absent due to flattening and compression caused by cerebrospinal fluid within the arachnoid mater filling this space, this phenomenon is typically observed in brain imaging (7). It can be classified as either primary (with no identifiable cause) or secondary (resulting from pituitary injury, necrosis, or pharmacological therapy) (8). The coexistence of an empty sella turcica and thyroid hormone resistance (THR) is likely an incidental finding, Al Mohareb O et al. (9), presented a case of THR due to a beta-subunit mutation with a partially empty sella, but without any correlation between the two conditions (9). The present case poses a diagnostic challenge, and brain MRI is crucial to rule out pituitary malignancies that could affect the endocrine axis. Consequently, it is also important to assess all anterior pituitary hormones to rule out endocrinopathies such as hyperprolactinemia and hypopituitarism (10). However, radiological and biochemical follow-ups are still required to monitor any changes in this anatomical finding that could lead to other hormonal abnormalities.

#### 2.2.3 Levodopa pharmacokinetics: mechanisms and challenges in Parkinson’s disease.

Parkinson’s disease is characterized by the loss of dopaminergic transmission in the nigrostriatal pathway (11). Today, most patients with Parkinson’s disease may achieve long-term benefits in terms of impact on disability and quality of life with the regular use of levodopa. Many challenges have been faced with the development of this molecule; the travel pathway of oral levodopa from its uptake in the gastrointestinal tube to its transfer to the brain is complex (12).

Levodopa or L-dopa serves as an aromatic amino acid precursor of dopamine because it is unable to cross the blood brain barrier (BBB) due to its molecular electrical charge (13). The uptake of L-dopa occurs in a short portion of the duodenum and proximal jejunum using sodium-dependent L-neutral amino acid transporters in the gut wall, along with other L-amino acids from dietary sources (14, 15). A vast majority of orally administered L-dopa is cleared by first-pass hepatic metabolism; unless catecholamine-O-methyltransferase (COMT) inhibitors are used, higher amounts of L-dopa would remain in the bloodstream. Blocking levodopa metabolism through its travel from the gut to the brain with COMT inhibitors such as tolcapone and entacapone would help increase its time plasma concentration curve and extend the wearing-off effect. Drugs that inhibit systemic L-AAAD (left aromatic amino acid decarboxylase) activity are useful but limited to the extent of their actions (16, 17).

L-dopa is transported across the BBB by LAT1, a large neutral amino acid transporter expressed in the endothelial cells. However, it is also apparent that levodopa has a localized effect on the neurovascular unit, which comprises neurons, astrocytes, and endothelial cells (18, 19). Upon crossing the blood-brain barrier, L-dopa is decarboxylated to dopamine and stored in monoaminergic presynaptic terminals in nigrostriatal pathways (13).

#### 2.2.4 Dopamine effects on thyroid activity

Moreover, discussing the dopamine effects on endocrine function, this molecule inhibits the synthesis of prolactin in the pituitary gland, being convenient for the treatment of prolactinomas. Furthermore, TRH activates the presynaptic dopaminergic neurons, thereby increasing the release of this neurotransmitter. *In vitro* models have shown an improvement in motor symptoms related to Parkinson’s disease. Additionally, dopamine hinders thyroid hormone release mediated by adrenergic and dopaminergic receptors and TSH, although the exact mechanism remains unknown (20)​. It has also been found that dopamine decreases free alpha subunit and TSH beta in *in vitro* models (21). Furthermore, the dopamine precursor levodopa has been associated with changes in thyroid function, particularly by decreasing TSH levels (22)​. In regard to the effect of dopamine in the pituitary, it has been described how infusions with this neurotransmitter caused a decrease in TSH concentration in six volunteers (23). The levels remained suppressed until the infusion was paused, where the TSH levels increased above the normal range before returning to a normal concentration. This suggests that the effect of levodopa in the release of TSH is temporarily while the medication is administered. The outcome was also described for prolactin, LH, FSH in a similar manner, but TSH and prolactin were suppressed in a higher proportion. A comparable result was found with the use of levodopa/carbidopa in patients with Parkinson’s disease (23). This is thought to occur by a direct action of dopamine in thyrotropic cells (24). The effect has been reported to be more pronounced in patients aged 64 and older (25). Similarly, other studies have established a decreased effect of TRH on TSH release when dopamine is administered (26). This was also concluded by Rabey et al., in patients with Parkinson´s disease who were treated chronically with L-dopa. Nevertheless, they did not find a change on basal T3 or T4 levels in this population (27). Conversely, other studies have not found a relationship between the use of this medication and changes in TSH concentration (28)​. Therefore, there is still controversy regarding these outcomes. Concerning the present case, it is believed that the administration of levodopa along with the variable adjustment of levothyroxine aided in the normalization of TSH and total T3 levels in this patient with a suspected THR, assisting in the standardization of the concentration of these hormones in blood and thus allowing to carry out the resection of the neoplastic remnant. Larger studies regarding the effect of levodopa in patients with suspected THR are still required to assess the role of this medication in individuals without Parkinson´s disease.

#### 2.2.5 Effects of other PD medications on thyroid activity

Concerning other possible effects of PD medications on the hypothalamic-pituitary-thyroid axis, it has been described in rat models that the administration of monoamine oxidase A inhibitors can decrease the thyroid uptake of iodide by an indirect mechanism. Nevertheless, this was not concluded when monoamine oxidase B inhibitors were administered (29). In addition, bromocriptine has been reported to be used in a patient with THR. Acutely, the dopamine agonist reduced TSH and prolactin levels. Continuous administration of the medication reduced the patient´s goiter and symptoms, suppressed TSH levels and reduced T4 concentrations to almost a normal range. T3 values were not significantly reduced. Nonetheless, when bromocriptine was discontinued, the clinical manifestations and hormone levels increased (30). Other studies have described a decline in TSH and also T3 levels in patients with THR who have received bromocriptine (31). This dopamine agonist suggests a similar effect on the thyroid axis similar to the effect levodopa has had on the current case which may be useful in patients with THR that have untreatable symptoms or who need a normalization of the TSH values for surgery as our patient.

## 3 Conclusion

Overall, in the present case with probable THR, the use of levodopa/carbidopa for the treatment of Parkinson’s disease along with the titration of levothyroxine allowed for the normalization of TSH and total T3 levels. This role was essential to perform the surgical resection of the residual tumoral fragment. Finally, it is believed that the underlying mechanism that allowed for this adjustment was throughout the influence of dopamine in the decrease in TSH beta and free alpha subunit.

## Data Availability

The original contributions presented in the study are included in the article/supplementary material. Further inquiries can be directed to the corresponding author.
